# Molecular Signals in Nodulation Control

**DOI:** 10.3390/ijms18010125

**Published:** 2017-01-10

**Authors:** Peter M. Gresshoff, Brett J. Ferguson

**Affiliations:** Centre for Integrative Legume Research, The University of Queensland, St Lucia, Brisbane Qld 4072, Australia

Our world is facing major problems relating to food production. According to an August 30, 2015 program of LANDLINE (Australian Broadcasting Corporation, Australia), we lose 120,000,000 hectares of agricultural land per year due to population growth, associated urbanisation, and desertification. The expected population of >10 billion human inhabitants of this planet by 2050 (only 34 years away!) will require an increase of 46% in staple grain production and about 76% in animal protein output. These goals may be difficult to achieve with predicted climate changes, sociological changes towards higher consumption, political and military unrest, etc.

For these reasons, it is essential that we now focus biological research at increased productivity with increased sustainability. One aspect of plant production is the absolute requirement for nitrogen. It is a key element, next to carbon, in most biological molecules (such as metabolites, proteins and even nucleic acids). In the past it was possible to recycle waste to supply this nitrogen need. However, large-scale agriculture now relies on supplementation of field sites with industrial fertilisers including those providing nitrogen (such as various nitrate, urea, and ammonium formulation). This practice is NOT sustainable and not economically advantageous, as the increased yield is burdened by: (1) the costs of energy-demanding production of the Haber–Bosch process; (2) environmental negatives such as nitrous oxide (a strong Greenhouse gas 296 times as effective as CO_2_) release; and (3) surface and ground water nitrification, leading to eutrophication (as an example see the Mississippi River Delta in the Gulf of Mexico). 

Alternatives of nitrogen supply are clearly needed. One of these is the natural process of root nodulation and associated symbiotic nitrogen fixation as seen agriculturally in many legume crops, such as soybean, chickpea, clovers, medics, peas, peanuts, and even trees, such as acacia, robinia, and the biodiesel feedstock tree pongamia (Gresshoff et al. 2015) [1]. This process occurs in de novo root organs called nodules, which are induced by a range of soil bacteria, broadly called “rhizobia”. These organised structures then provide the “prison” for the invading bacterium, so that the correct physiological conditions such as ample plant energy supply and restricted, but stable, oxygen concentration are achieved (Ferguson et al. 2010) [2].

Extensive international research on multiple levels of science (ranging from ecology and soil science to plant physiology and genomics) on nodulation and nitrogen fixation over the last century has provided amazing insights and understanding of these processes; this in turn has improved agricultural yield and lowered inputs. One only needs to look at the development of soybean cropping in Brazil. Low and inconsistent yields half a century ago are now replaced by a robust and competitive industry. However, like any biological process, there are genetic and environmental factors that control the outcome. There are legume and bacterial mutants that fail to nodulate all together; some nodulate but fail to fix nitrogen. There are acidic or nitrate-rich soils that suppress nodulation and, thus, the symbiotic input.

We now know that the symbiosis is facilitated by a complex signal interchange between plant and bacterium and in reverse (Ferguson and Mathesius 2014) [3]. For example, we know that in legume nodulation induction the plant root secretes flavones and isoflavones into the rhizosphere which specifically induce rhizobia nodulation-genes (NOD and NOL) that control the synthesis of a critical bacterial signal called the Nod-factor. This molecule is perceived by legume plants through a root-membrane localised Nod-factor receptor duplex made up of two LysM-type receptor kinases. We know that subsequent calcium movement in the host cells cause developmental responses such as the initiation of cell divisions in both cortex and pericycle as well as major deformations of root hair extension, leading to bacterial invasion.

We know that initiated cell division clusters in the root regulate the future development of such clusters. This process is called Autoregulation of Nodulation and amazingly is facilitated by a feedback loop involving root to shoot and shoot to root signaling (Reid et al. 2011) [4]. Short peptides (related to the non-symbiotic CLAVATA3 peptide first discovered in Arabidopsis) are synthesised in Rhizobium-induced cell clusters of the root and travel to the shoot (Hastwell et al. 2015) [5], where they interact with the Leucine-Rich Repeat (LRR) receptor kinase called NARK, SUNN or HAR1 (orthologues receptors with different names in different legume species, but ultimately the same!). Activation of this receptor leads to the suppression of a specific microRNA species, which is phloem-translocated to the root, causing a suppression of further nodulation induction. Numerous questions about these and other factors remain.

Positively one finds that the analysis of the nodulation induction and regulation processes leads to insights into plant development and associated biochemistry in general. Indeed, research over the last decade has yielded insight into these, using approaches ranging from agronomic field trials to molecular genetic and genomic technologies. This Special Issue of the *International Journal of Molecular Sciences* (*IJMS*) is thus devoted to recent advances in the field of “Molecular Signals in Nodulation Control” [6].

In this Special Issue [6], a series of studies are presented (five research papers and two reviews), that cover different aspects of legume nodulation. Formey et al. (2016) identify exciting new microRNAs and their corresponding targets in *Phaseolus vulgaris* that may function in the regulation of early nodulation events [7]. Using *Medicago truncatula*, van Noorden et al. (2016) elegantly established that high levels of nitrate appear to inhibit nodulation via multiple pathways, including changes to flavonoid metabolism, defense responses and redox changes, but not root hair curling [8]. Interestingly, the authors also established that auxin accumulation in response to rhizobia still occurs in the presence of nitrate, but does not localize to the nodule initiation sites. Furthermore, expression of auxin efflux-encoding *PIN* genes in *M. truncatula* was analysed by Sanko-Sawczenko et al. (2016) with differences established between nodules and root tips [9]. Lardi et al. (2016) report on their investigations into the metabolome of nodules and roots from four different *B. diazoefficiens*-infected host plants, in addition to soybean nodules harvested at different time points or having been infected by rhizobia strains mutated in key nitrogen-fixation genes [10]. Moreover, Siczek and Lipiec (2016) show that the functional diversity of microbial communities was a less sensitive tool than enzyme activities in assessing rhizobial inoculation on microbial activity in the rhizosphere of faba bean [11]. Collectively, these research articles advance the molecular understanding and potential impact of legume nodulation via the assessment of different aspects of the process.

In addition to the abovementioned research articles, the Special Issue of IJMS includes two in depth review articles pertaining to “Molecular Signals in Nodulation Control”. López-Baena provide an exceptionally thorough overview of the key bacterial signals involved in the legume–rhizobia symbiosis [12]. This includes nodulation factors, different surface polysaccharides (exopolysaccharides, lipopolysaccharides, cyclic glucans, and K-antigen capsular polysaccharides), and effectors delivered via a type 3 secretion system. The authors focus their review on *S. fredii*, a rhizobia species having a broad host range that includes important agricultural crops which form either determinate or indeterminate nodule structures. The review by Montiel et al. (2016) focuses on the highly important and versatile roles of respiratory burst oxidase homologues (RBOH) in the legume–rhizobia symbiosis [13]. These enzymes of the plasma membrane are dedicated to the production of reactive oxygen species (ROS) that act in the development and control of legume nodules. Indeed, the authors highlight the role of RBOH-mediated ROS production at different stages of nodulation, indicating a function for ROS in both the regulation of rhizobial invasion and in nodule senescence.

It is hoped that advances reported in this Special Issue help to increase the efficiency of the legume symbiosis, with the aim of providing additional molecular tools to resolve the anticipated problems associated with future food production.
